# Supplementary material for: Comparison of Drying Techniques for Extraction of Bioactive Compounds from Olive-Tree Materials
Source: Foods. 2023 Jul 12;12(14):2684. doi: 10.3390/foods12142684 (PMC10379223; doi:10.3390/foods12142684)
Supplement: Supplementary file 1 [file foods-12-02684-s001.zip › foods-2474859-supplementary/Supplementary table.pdf]

Supplementary Table S1. Parameters for identification of bioactive compounds in olive-tree materials.

Compound name	R_t	Formula	Precursor ion (*m/z*)	Main product ions (*m/z*)	Priority samples
**Secoiridoids**					
Oleuropein [a]	12.15	$C_{25}H_{32}O_{13}$	539.176	507.2762; 225.0747; 112.9845	Leaves
Oleuropein glucoside	11.37	$C_{31}H_{42}O_{18}$	701.2285	539.1776; 275.0893; 153.0529	Leaves
Oleuropein quinone	12.41	$C_{25}H_{30}O_{13}$	537.1612	223.0601; 151.0396; 110.1298	Leaves
Ligstroside	13.05	$C_{25}H_{32}O_{12}$	523.1813	291.0846; 259.0948; 137.0594	Leaves and olive pomace
Hydroxyoleuropein	10.70	$C_{25}H_{32}O_{14}$	555.1709	537.1597; 291.0864; 151.0380	Leaves
Oleacein [a]	12.39	$C_{17}H_{20}O_6$	319.1175	153.0440; 69.0341; 59.0129	Leaves and olive pomace
Oleaceinic acid	10.49	$C_{17}H_{19}O_7$	335.1113	199.0631; 181.0715; 151.0385	Olive pomace
Oleocanthal [a]	13.49	$C_{17}H_{20}O_5$	303.1228	181.0051; 137.0585; 69.0331	Olive pomace
Oleocanthalic acid	12.61	$C_{17}H_{20}O_6$	319.1185	183.0654; 139.0745; 69.0343	Olive pomace
Oleuropein aglycone [a]	14.11	$C_{19}H_{22}O_8$	377.1233	275.0556; 153.0494; 59.0128	Leaves
Oleuropein aglycone quinone	14.01	$C_{19}H_{20}O_8$	375.1095	275.0553; 153.0560; 59.0128	Olive pomace
Ligstroside aglycone [a]	14.59	$C_{19}H_{22}O_7$	361.1282	291.0870; 139.0402; 111.0072	Olive pomace
2-methoxyoleuropein	12.48	$C_{26}H_{34}O_{14}$	569.1874	537.1590; 403.1233; 151.0386	Olive pomace
Demethyloleuropein aglycon	12.93	$C_{18}H_{20}O_8$	363.1085	229.1074; 121.0666; 59.0123	Leaves
Hydroxyoleuropein aglycon	12.27	$C_{19}H_{22}O_9$	393.1182	291.0503; 151.0399; 62.9845	Olive pomace
GL3 [a]	12.14	$C_{48}H_{64}O_{27}$	1071.356	909.3060; 685.2386; 101.0248	Olive pomace
Nuzhenide [a]	12.81	$C_{31}H_{42}O_{17}$	685.2338	685.2447; 453.1346; 101.0232	Olive pomace

Hydroxylated form of decarboxymethyl oleuropein aglycone	10.49	$C_{17}H_{20}O_7$	335.1124	202.9119; 151.03770; 69.0350	Olive pomace
**Simple phenols**					
3-methylcatechol [a]	9.42	$C_7H_8O_2$	123.0441	95.0479; 69.0329; 41.0015	Leaves and olive pomace
Hydroxytyrosol [a]	9.43	$C_8H_{10}O_3$	153.0544	123.0444; 93.0329; 44.9976	Leaves and olive pomace
Hydroxytyrosol glucoside	8.89	$C_{14}H_{20}O_8$	315.1072	153.0537; 123.0442; 59.0129	Olive pomace and leaves
Hydroxytyrosol-lathyroside	8.99	$C_{19}H_{28}O_{12}$	447.1491	315.888; 153.0548; 44.9976	Leaves and olive pomace
Tyrosol acetate	10.48	$C_{10}H_{12}O_3$	179.0705	137.3739; 123.0421; 68.9950	Olive pomace and olive
**Flavonoids**					
Apigenin [a]	14.49	$C_{15}H_{10}O_5$	269.0441	225.0538; 151.0028; 117.0333	Leaves
Luteolin [a]	13.92	$C_{15}H_{10}O_6$	285.0395	199.0389; 175.0396; 151.0033	Leaves
Luteolin-7-O-glucoside [a]	12.69	$C_{21}H_{20}O_{11}$	447.0921	327.0486; 285.0405; 167.0329	Leaves
Luteolin-7-rutinoside [a]	10.90	$C_{27}H_{30}O_{15}$	593.1505	285.0402; 151.9935; 137.8169	Leaves
Quercitrin [a]	12.26	$C_{21}H_{20}O_{11}$	447.0920	300.0264; 254.9830; 151.0364	Leaves
Quercetin-3-glucoside [a]	11.46	$C_{21}H_{20}O_{12}$	463.0875	424.4496; 300.0259; 190.9303	Leaves
Rhoifolin [a]	11.54	$C_{27}H_{30}O_{14}$	577.1526	269.0449; 151.0037; 112.9837	Leaves
Rutin [a]	11.14	$C_{27}H_{30}O_{16}$	609.1456	385.1314; 301.0322; 151.0041	Leaves
**Triterpenes**					
Maslinic acid [a]	16.67	$C_{30}H_{48}O_4$	471.3469	392.9793; 266.9875; 118.9934	Olive pomace, leaves and olive
Oleanolic acid	17.51	$C_{30}H_{48}O_3$	455.3524	396.9865; 187.0260; 112.9804	Leaves

R_t, Retention time.
[a]Confirmed by analytical standard.
