# Supplementary figures and images for: Comparison of Drying Techniques for Extraction of Bioactive Compounds from Olive-Tree Materials
Source: Foods. 2023 Jul 12;12(14):2684. doi: 10.3390/foods12142684 (PMC10379223; doi:10.3390/foods12142684)

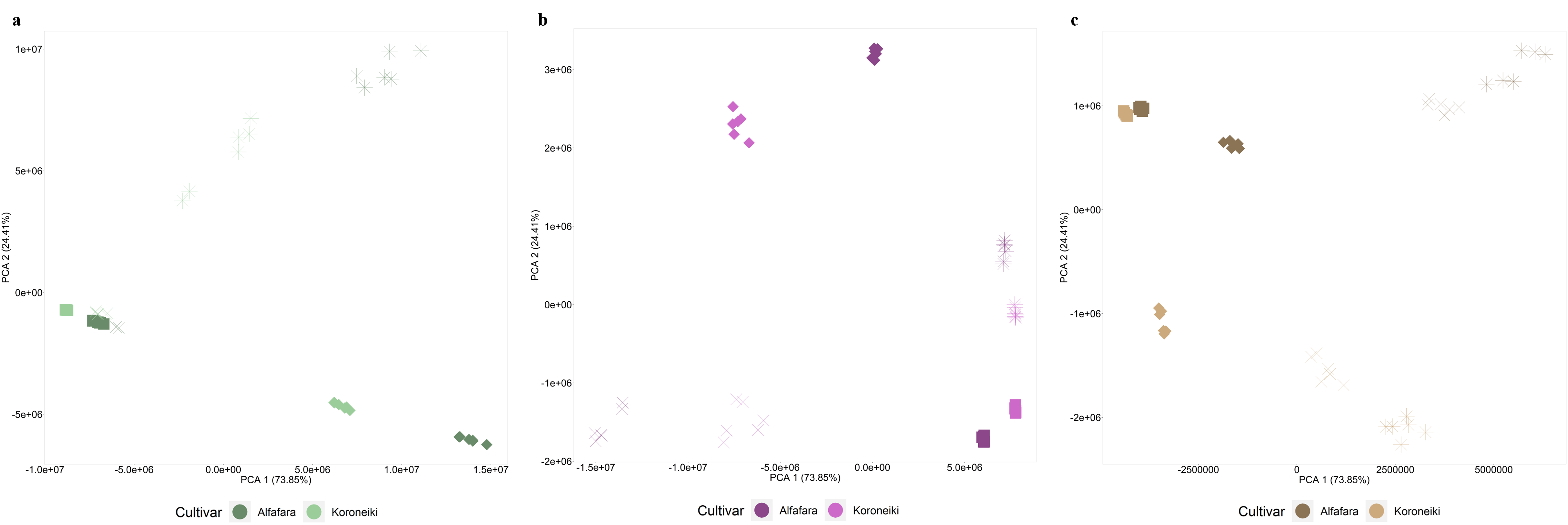

Supplement: Supplementary file 1 [file foods-12-02684-s001.zip › foods-2474859-supplementary/Figure S1.tif]

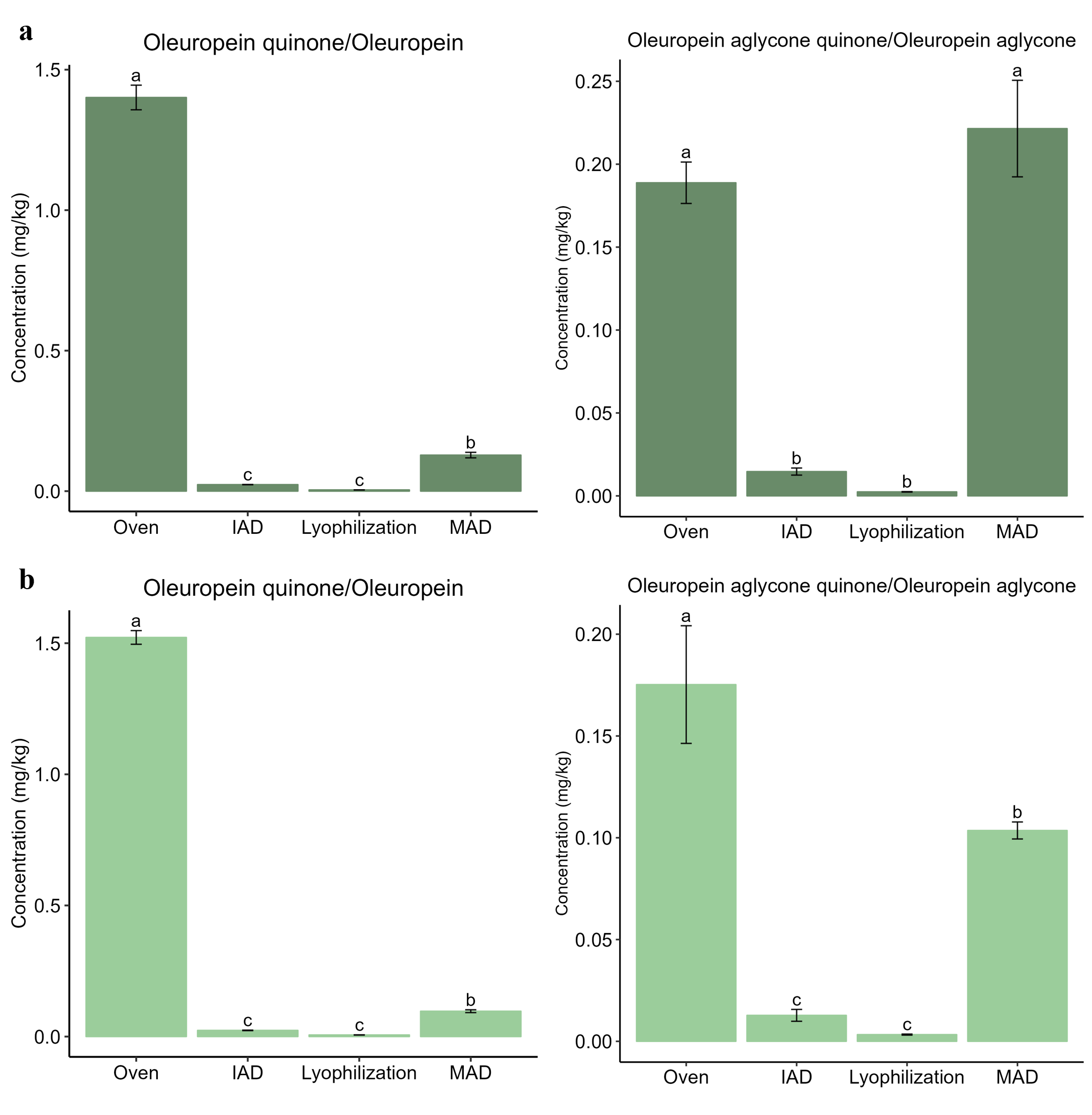

Supplement: Supplementary file 1 [file foods-12-02684-s001.zip › foods-2474859-supplementary/Figure S2.tif]

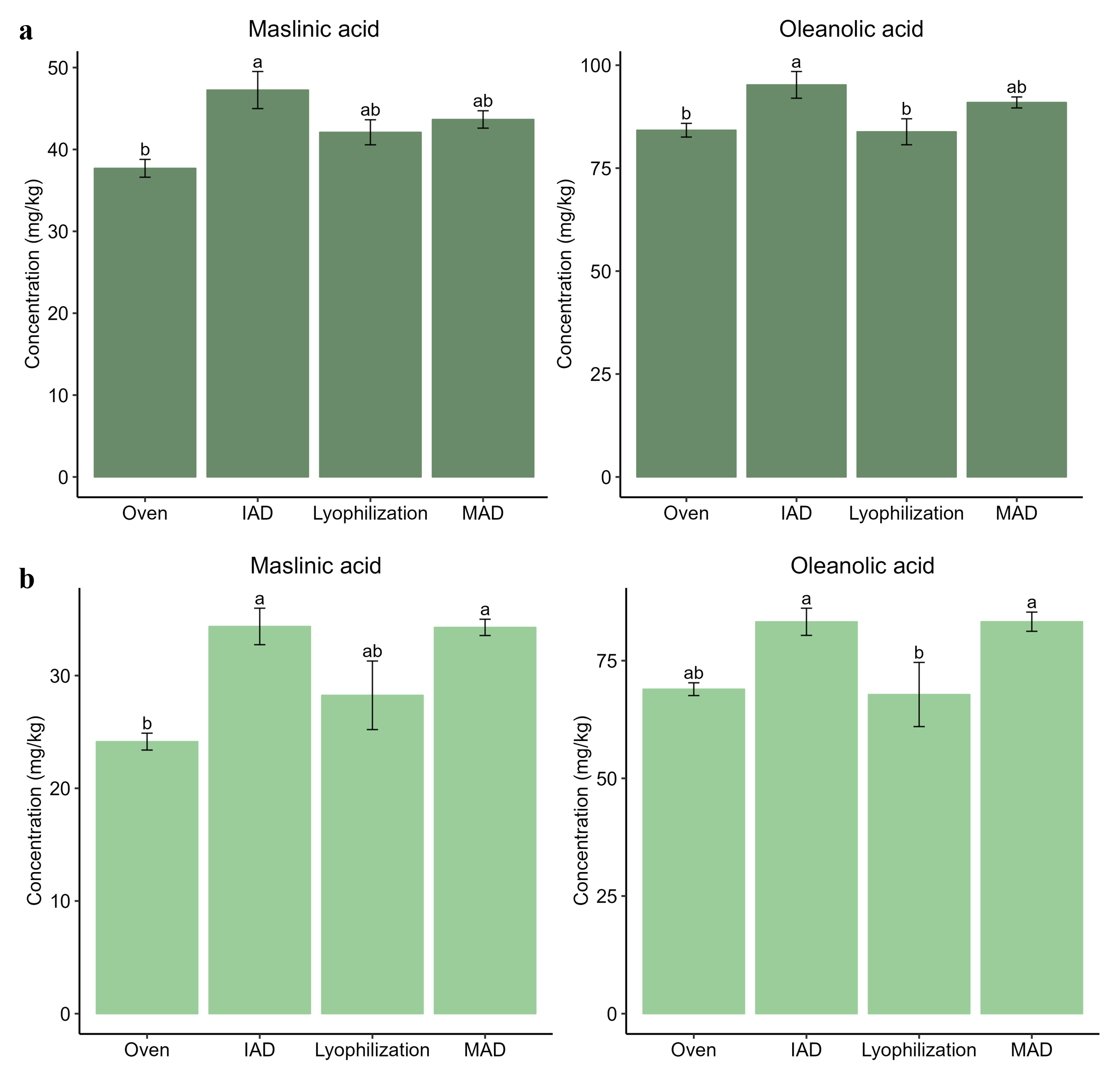

Supplement: Supplementary file 1 [file foods-12-02684-s001.zip › foods-2474859-supplementary/Figure S3.tif]

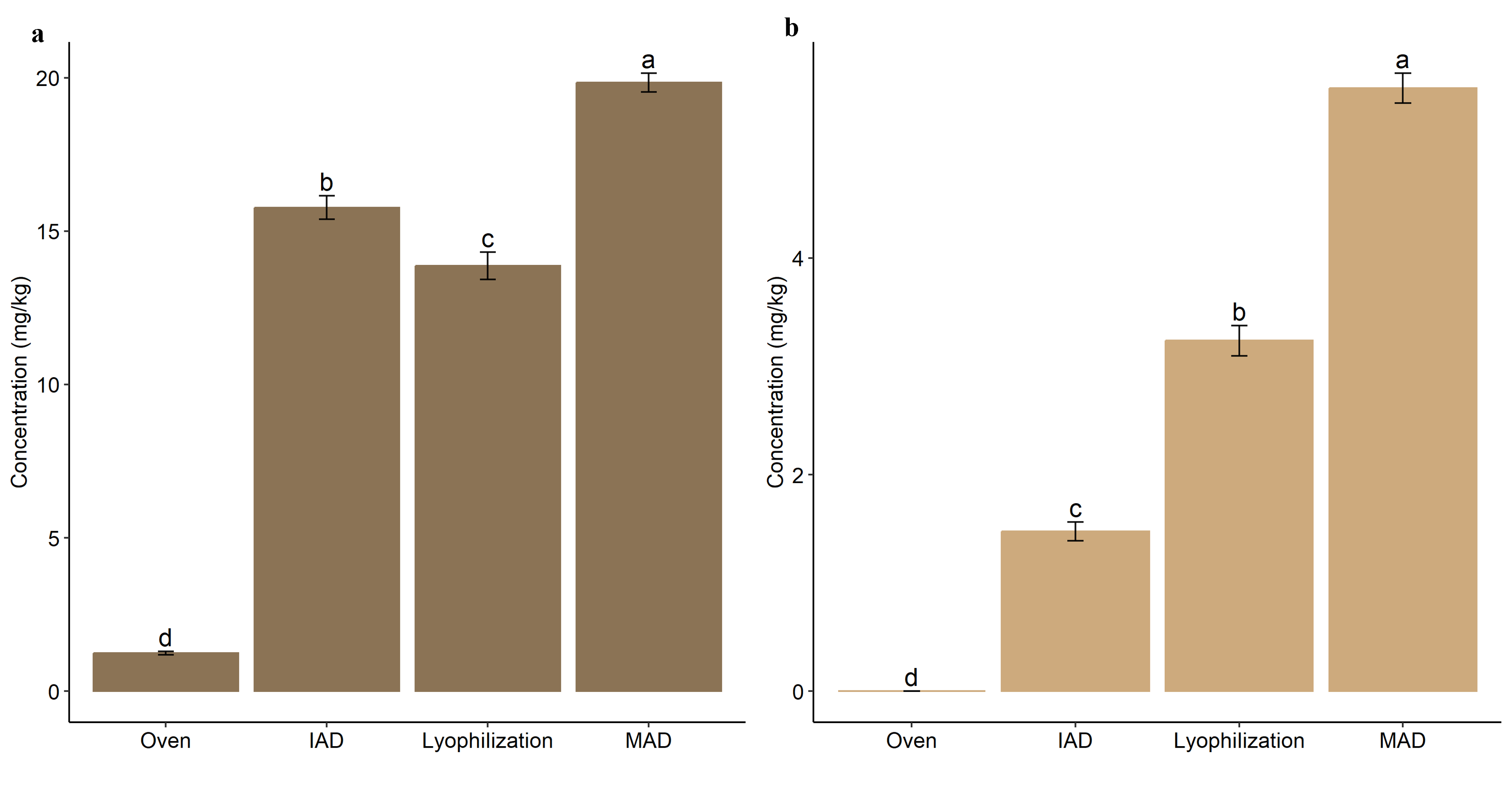

Supplement: Supplementary file 1 [file foods-12-02684-s001.zip › foods-2474859-supplementary/Figure S4.tif]

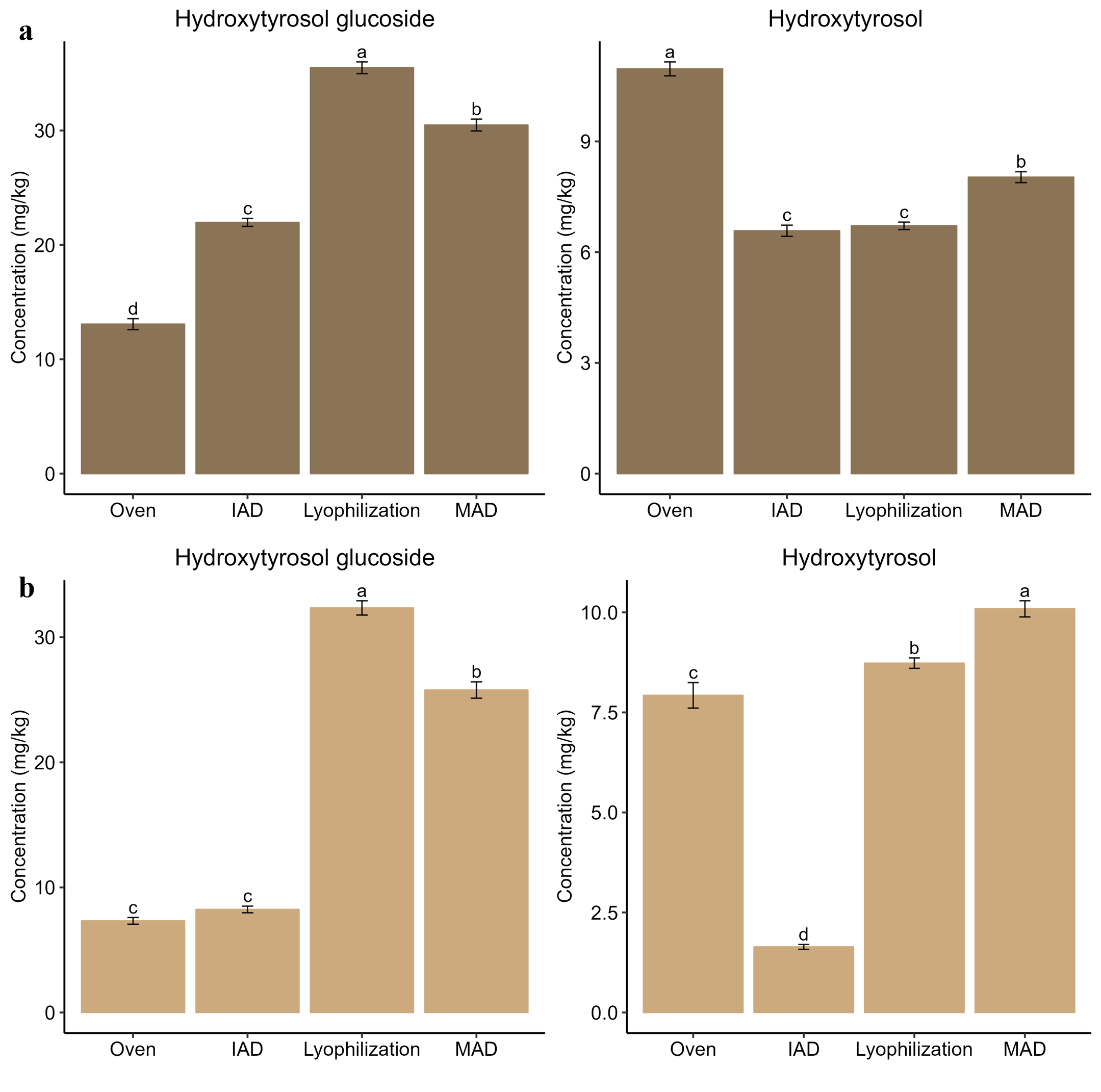

Supplement: Supplementary file 1 [file foods-12-02684-s001.zip › foods-2474859-supplementary/Figure S5.tif]

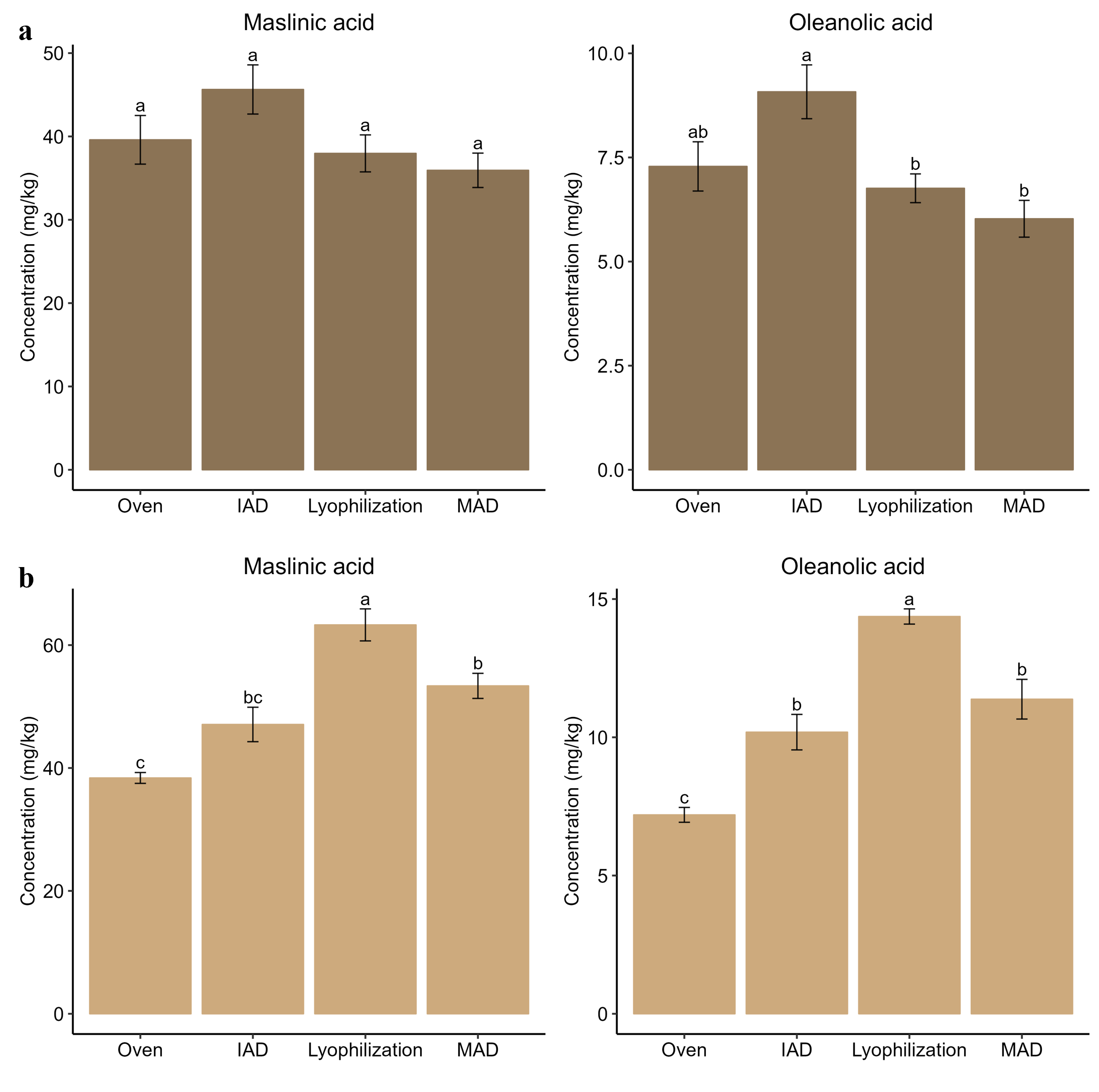

Supplement: Supplementary file 1 [file foods-12-02684-s001.zip › foods-2474859-supplementary/Figure S6.tif]

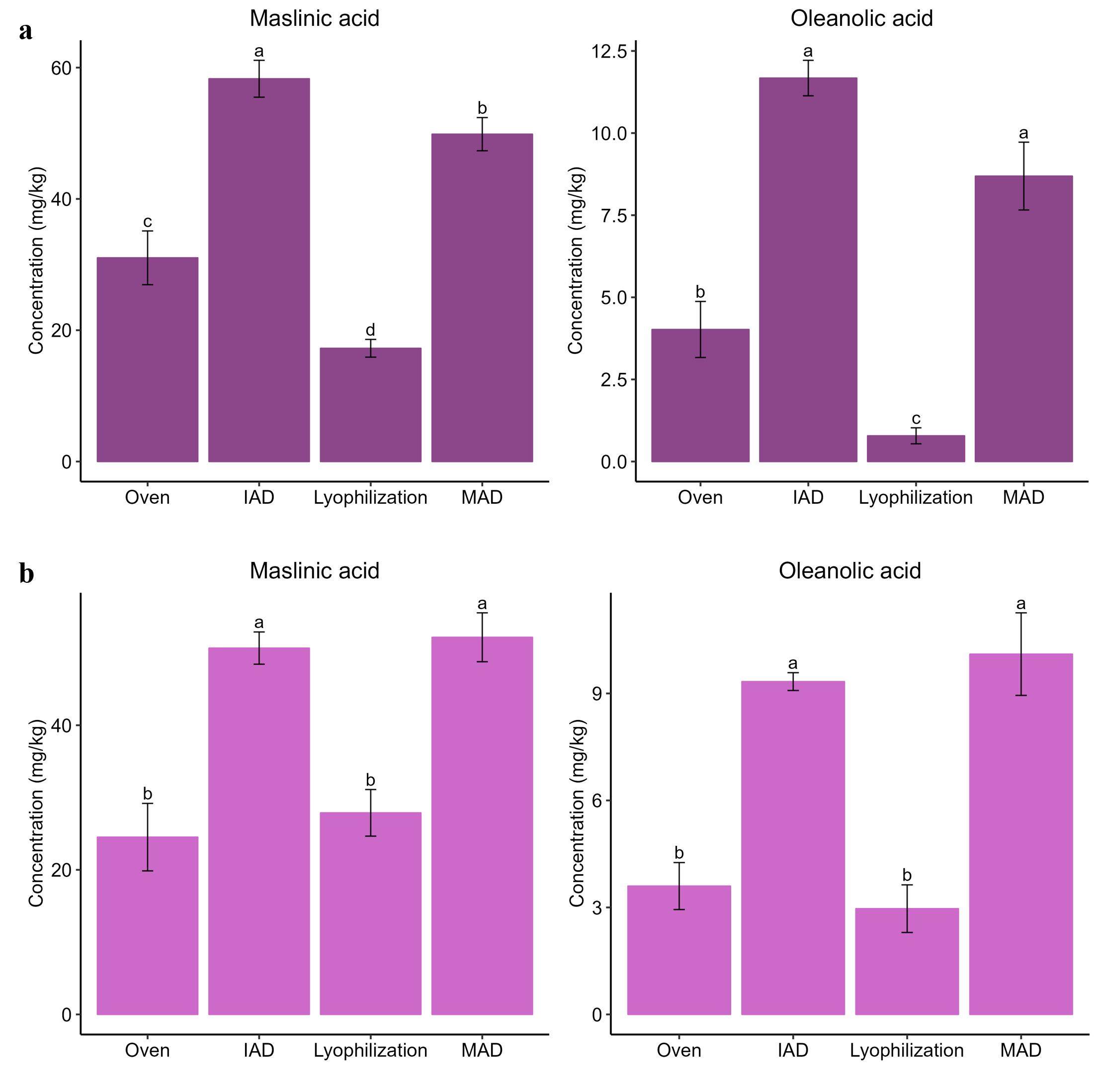

Supplement: Supplementary file 1 [file foods-12-02684-s001.zip › foods-2474859-supplementary/Figure S7.tif]

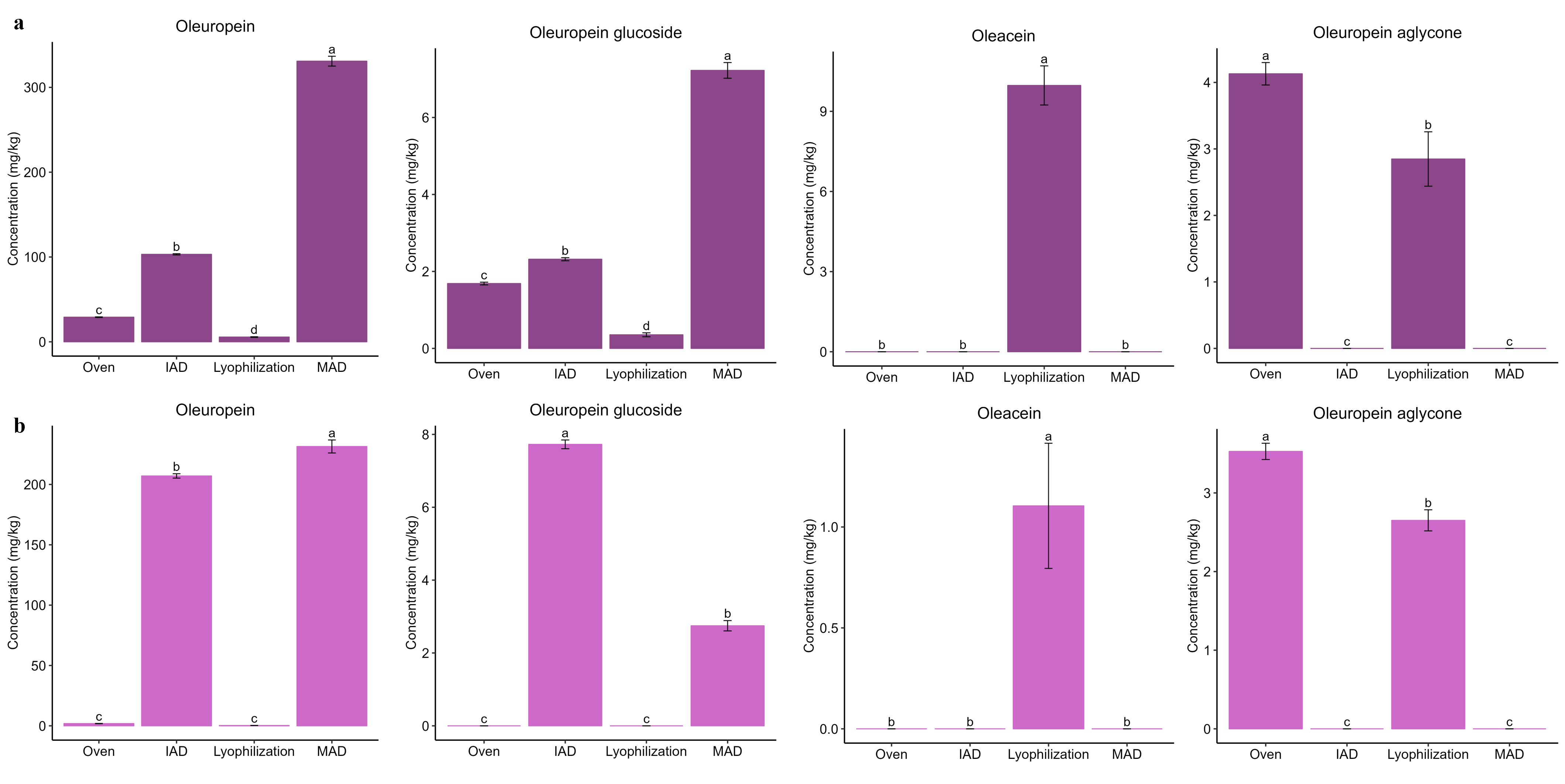

Supplement: Supplementary file 1 [file foods-12-02684-s001.zip › foods-2474859-supplementary/Figure S8.tif]
